# Lipidomic Profiling Identifies Serum Lipids Associated with Persistent Multisite Musculoskeletal Pain

**DOI:** 10.3390/metabo12030206

**Published:** 2022-02-25

**Authors:** Canchen Ma, Ming Liu, Jing Tian, Guangju Zhai, Flavia Cicuttini, Yvette L. Schooneveldt, Peter J. Meikle, Graeme Jones, Feng Pan

**Affiliations:** 1 Menzies Institute for Medical Research, University of Tasmania, Private Bag 23, Hobart, TAS 7000, Australia; canchen.ma@utas.edu.au (C.M.); j.tian@utas.edu.au (J.T.); graeme.jones@utas.edu.au (G.J.); 2 Division of Biomedical Sciences (Genetics), Faculty of Medicine, Memorial University of Newfoundland, St. John’s, NL A1C 5S7, Canada; ming.liu@med.mun.ca (M.L.); guangju.zhai@med.mun.ca (G.Z.); 3 Department of Epidemiology and Preventive Medicine, School of Public Health and Preventive Medicine, Monash University, 553 St Kilda Road, Melbourne, VIC 3004, Australia; flavia.cicuttini@monash.edu; 4 Baker Heart and Diabetes Institute, Melbourne, VIC 3004, Australia; yvette.schooneveldt@baker.edu.au (Y.L.S.); peter.meikle@baker.edu.au (P.J.M.); 5 Faculty of Medicine, Nursing and Health Sciences, Monash University, Melbourne, VIC 3004, Australia

**Keywords:** persistent multisite musculoskeletal pain, lipidomics, biomarker, lipid species, lipid classes

## Abstract

Lipid mediators have been suggested to have a role in pain sensitivity and response; however, longitudinal data on lipid metabolites and persistent multisite musculoskeletal pain (MSMP) are lacking. This study was to identify lipid metabolic markers for persistent MSMP. Lipidomic profiling of 807 lipid species was performed on serum samples of 536 participants from a cohort study. MSMP was measured by a questionnaire and defined as painful sites ≥4. Persistent MSMP was defined as having MSMP at every visit. Logistic regression was used with adjustment for potential confounders. The Benjamini–Hochberg method was used to control for multiple testing. A total of 530 samples with 807 lipid metabolites passed quality control. Mean age at baseline was 61.54 ± 6.57 years and 50% were females. In total, 112 (21%) of the participants had persistent MSMP. Persistent MSMP was significantly associated with lower levels of monohexosylceramide (HexCer)(d18:1/22:0 and d18:1/24:0), acylcarnitine (AC)(26:0) and lysophosphatidylcholine (LPC)(18:1 [sn1], 18:2 [sn1], 18:2 [sn2], and 15-MHDA[sn1] [104_sn1]) after controlling for multiple testing. After adjustment for age, sex, body mass index, comorbidities, and physical activity, HexCer(d18:1/22:0 and d18:1/24:0) and LPC(15-MHDA [sn1] [104_sn1]) were significantly associated with persistent MSMP [Odds Ratio (OR) ranging from 0.25–0.36]. Two lipid classes—HexCer and LPC—were negatively associated with persistent MSMP after adjustment for covariates (OR = 0.22 and 0.27, respectively). This study identified three novel lipid signatures of persistent MSMP, suggesting that lipid metabolism is involved in the pathogenesis of persistent pain.

## 1. Introduction

Musculoskeletal pain is the most common complaint in the general population and the leading cause of years lost to disability [1,2]. An estimated prevalence of 20% to 40% was reported globally with foreseen increasing individual and societal burden in the next decades [3,4]. A clinically relevant entity of multisite musculoskeletal pain (MSMP) has been defined, as musculoskeletal pain rarely occurs at single site [5]. More detrimental health outcomes associated with MSMP have been reported, such as health-related quality of life, fractures and sleep disturbance [6,7,8,9,10]. However, the initiation and persistence of MSMP is far from being fully understood.

Alteration of metabolic pathways has been suggested to be involved in the pathogenesis of musculoskeletal pain [11,12]. Metabolites are intermediates and end products of cellular regulatory processes and have critical roles in metabolic pathway regulation and signal transductions [11,13,14]. Metabolomics analysis has been used to identify and quantify metabolites, some of which are found to be implicated in the pathogenesis of musculoskeletal pain [15].

It has been suggested that endogenous lipid species as mediators have essential roles in pain severity and response [16,17,18,19,20]. Our recent study identified and replicated one lipid (i.e., sphingomyelin (SM) C18:1) associated with the presence of MSMP in two independent cohorts [21]. In addition, two proinflammatory lipid compounds in association with the presence of MSMP were also identified [22]. This may reflect that dysfunction in lipid metabolism is involved in the MSMP initiation. However, no longitudinal study has examined the roles of lipid mediators in MSMP persistence. Therefore, this study aimed to identify lipid signatures associated with persistent MSMP by using lipidomics.

## 2. Results

Among the 536 serum samples profiled, 530 passed quality control (QC) and were included in the subsequent analyses. There were 195, 201 and 215 participants reporting MSMP at 2.6-year (37%), 5.1-year (38%) and 10.7-year (41%) follow-ups, respectively. In total, 112 participants had persistent MSMP (21%) and 418 had non-persistent MSMP (79%). The persistent MSMP group had more females (67% vs. 46%), and was less physically active (7234.72 ± 3009.63 vs. 8034.60 ± 3299.35 steps per day) (Table 1) than the non-persistent MSMP group. The persistent MSMP group had higher prevalence of osteoarthritis (OA) (65% vs. 30%) and emphysema (7% vs. 2%) than the non-persistent MSMP group. There was no difference in age, body mass index (BMI), and presence of rheumatoid arthritis (RA) or diabetes between the two groups (Table 1).

### 2.1. Lipid Markers and MSMP

In total, 850 ‘lipid measures’ representing 807 lipid species from 49 classes were quantified in all 530 serum samples and passed QC. A total of 53 lipid species and 5 lipid classes were found to be associated with MSMP with *p* < 0.05 (Supplementary Tables S1 and S2). Lipid species including SM(38:3) (a), SM(40:4), monohexosylceramide (HexCer) (d18:1/24:0) and acylcarnitine (AC) (26:0) were significantly associated with MSMP at 2.6-year follow-up after controlling multiple testing, but these associations became non-significant after adjusting for confounding factors (Table 2). The HexCer class was significantly associated with MSMP at 2.6-year follow-up, but this significant association did not hold after adjusting for confounding factors (Table 2). The ratio of lysophosphatidylcholine (LPC) to phosphatidylcholine (PC) was negatively associated with MSMP at 2.6-year follow-up (odds ratio (OR) = 0.33, 95% confidence interval (CI): 0.13, 0.85), but this association became non-significant after adjustment for confounding factors (OR = 0.74, 95%CI: 0.22, 2.45).

### 2.2. Lipid Markers for Persistent MSMP

**Figure 1:**
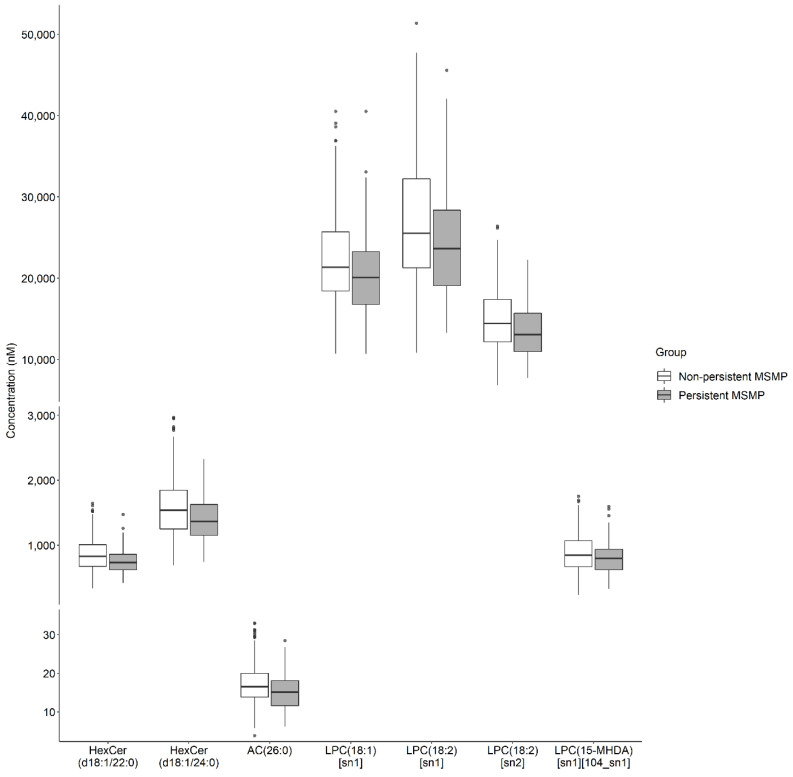
The original concentrations of serum lipid species in persistent MSMP and non-persistent MSMP groups. MSMP: multisite musculoskeletal pain; HexCer: monohexosylceramide; AC: acylcarnitine; LPC: lysophosphatidylcholine; MHDA: methylhexadecanoic acid. *p* values were obtained by independent sample Student’s *t*-test.

**Figure 2:**
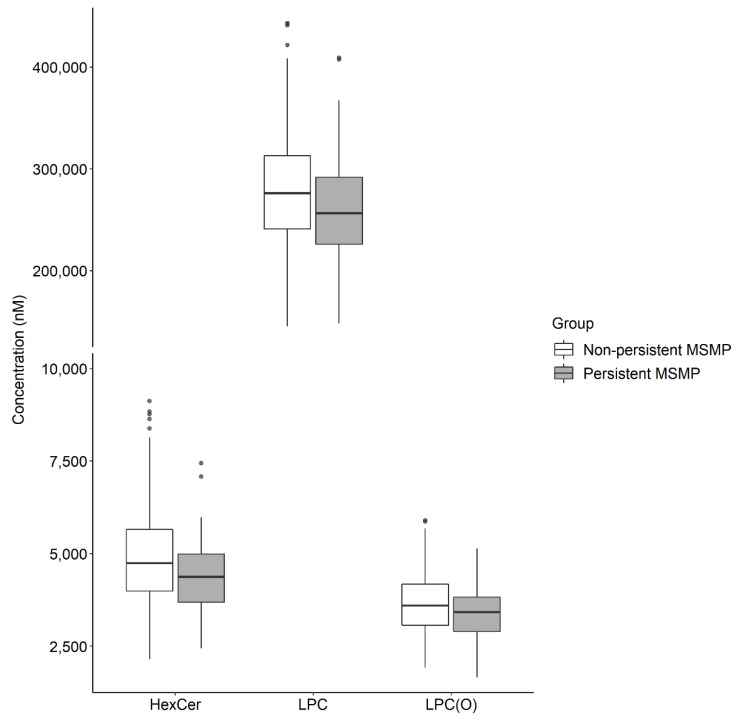
The original concentrations of lipid classes in persistent MSMP and non-MSMP groups. MSMP: multisite musculoskeletal pain; HexCer: monohexosylceramide; LPC: lysophosphatidylcholine; LPC(O): lysoalkylphosphatidylcholine. *p* values were obtained by independent sample Student’s *t*-test.

In total, 107 lipid species were found to be associated with persistent MSMP with *p* < 0.05 (Supplementary Table S3). After controlling multiple testing, 7 lipid species, i.e., HexCer (d18:1/22:0 and d18:1/24:0), AC 26:0 and LPC (18:1 [sn1], 18:2 [sn1], 18:2 [sn2] and 15-MHDA [sn1] [104_sn1]), were found to be negatively associated with persistent MSMP, that is, participants with persistent MSMP had lower levels of these lipid species compared to those without persistent MSMP (Figure 1). Per log nM decrease in these lipid species was associated with a higher risk of persistent MSMP (Table 3). The significances remained for HexCer d18:1/22:0 and d18:1/24:0 and LPC 15-MHDA [sn1] [104_sn1] after adjustment for age, sex, BMI, physical activity and presence of any comorbidities (OR ranged from 0.25 to 0.36, Table 3). Seven lipid classes were associated with persistent MSMP with *p* < 0.05 (Supplementary Table S4). After controlling multiple testing, 3 lipid classes, HexCer, LPC, and lysoalkylphosphatidylcholine (LPC(O)), were negatively associated with persistent MSMP (Figure 2). Per log nM decrease in these lipid classes was also associated with a higher risk of persistent MSMP (Table 4). The significances remained for HexCer and LPC after adjustment for confounding factors (OR = 0.22 and 0.27, respectively, Table 4). The ratio of LPC to PC was negatively associated with persistent MSMP (OR = 0.14, 95%CI: 0.05, 0.44), but this association became non-significant after adjustment for confounding factors (OR = 0.32, 95%CI: 0.08, 1.30).

## 3. Discussion

This study is the first to identify that circulating serum levels of lipid markers are associated with persistent multisite musculoskeletal pain using lipidomics. We found that participants with persistent MSMP had lower levels of HexCer (d18:1/24:0 and d18:1/22:0), AC (26:0) and LPC (18:2 [sn2], 18:2 [sn1], 18:1 [sn1] and 15-MHDA [sn1] [104_sn1] compared to those with non-persistent MSMP. Decreased levels of three of these lipid species (i.e., HexCer d18:1/24:0 and d18:1/22:0, and LPC 15-MHDA [sn1] [104_sn1]) were significantly associated with a higher risk of persistent MSMP after controlling covariates and multiple testing. Furthermore, decreased levels of two lipid classes, HexCer and LPC, were significantly associated with a higher risk of persistent MSMP after controlling covariates and multiple testing. These results suggest that dysregulation of lipid metabolism is implicated in the persistence of pain.

Lipid mediators have been revealed to play bilateral roles in metabolic pathways and signaling transduction of pain [16]. However, only a few metabolomic profiling studies on musculoskeletal pain have been performed, all of which have been cross-sectional. Livshits et al. [23] identified that epiandrosterone sulfate was inversely associated with chronic widespread musculoskeletal pain in two cohorts, but the association did not hold after adjustment for fat mass index. In a case-control study including 22 fibromyalgia patients and 21 healthy controls, Caboni et al. [24] found an over-representation of LPC (14:0/0:0) and LPC (16:0/0:0) compounds in the group of fibromyalgia. There are, to date, two studies from our group identifying MSMP-associated metabolites. We found that two bioactive proinflammatory lipid compounds, LPC (26:0 and 28:1), were positively associated with MSMP using the extreme phenotype sampling strategy [22]. More recently, we identified and replicated the association between an elevated SM C18:1 level and the presence of MSMP in two independent cohorts using different criteria of MSMP [21]. In the current study, we observed that lipid species and class including SM(38:3) (a), SM(40:4), HexCer(d18:1/24:0) and AC(26:0) and the HexCer class were significantly associated with MSMP at 2.6-year follow-up after controlling multiple testing, but these associations became non-significant after adjusting for confounding factors. The discrepancy might be attributed to differences in studied population, MSMP definition and number of lipids included. The current study extended prior studies from cross-sectional to longitudinal analyses and found that the decreased serum levels of predominant lipid species (i.e., HexCer d18:1/22:0 and d18:1/24:0, and LPC 15-MHDA [sn1] [104_sn1]) and two lipid classes (i.e., HexCer and LPC) were associated with increased risk of persistent MSMP. Collectively, our findings together with previous studies indicate that dysfunction in lipid metabolism is not only implicated in the initiation of MSMP but also its persistence. However, differences in cross-sectional and longitudinal findings in this study suggest that lipids involved in the initiation and persistence of MSMP are different.

There are very limited studies that have examined the species and class of HexCex in pain conditions. Our finding that the level of HexCex was decreased in persistent MSMP is supported by a preclinical study [25] which reported a decreased level of HexCer in cancer-induced bone pain, which was characterized by the formation of peripheral neuropathic features at the site of bone tumor, and central neuropathic pathology in the dorsal horn and spinal glia. The HexCer class is one of the complex glycosphingolipids and abundant in biological membranes, particularly in the central nervous system. HexCer is generated from the central metabolite of sphingolipid metabolism (i.e., ceramide) by glycosylated synthase, and plays roles in bioactive pathways and signaling transductions [26]. The HexCer class includes glucosylceramide (GlcCer) and galactosylceramide (GalCer), which serve as precursors for the biosynthesis of more complex glycosphingolipids, such as gangliosides [27,28]. Gangliosides have roles in transferring nociceptive information from the periphery to the central nervous system (central sensitization) through the activation of neurotransmitter receptors or ion channels, such as transient receptor potential vanilloid type 1 (TRPV1) [29]. In addition, sphingosine synthesized from ceramide by ceramidase is a bioactive precursor of sphingosine 1-phosphate (S1P) [30,31], and the dysregulation of S1P pathway contributes to the establishment of central sensitization, which is a key step of developing chronic pain [32]. Although speculative, it is possible that the overactivation of ganglioside and S1P pathways might be implicated in persistent MSMP. The decreased level of the HexCer class in persistent MSMP may be directly due to the overactivation of the ganglioside pathway, resulting in overproduction of gangliosides. Alternatively, the overactivation of S1P pathway results in overproduction of sphingosine from ceramide, which may indirectly reduce the synthesis of HexCer.

The current study found that a lower level of LPC 15-MHDA [sn1] [104_sn1] was associated with persistent MSMP, which was contrary to prior studies on fibromyalgia and neuropathic pain. Hung et al. [33] reported that an increased level of LPC 16:0 in fibromyalgia patients modestly correlated with pain symptoms, and hyperalgesia in the mouse model. Rimola et al. [34] also reported elevated levels of LPC 16:0 and 18:1 in oxaliplatin-induced acute peripheral pain. This discrepancy may reflect different roles of lipid mediators in pain induction and maintenance, as numerous lipid mediators, including LPC, are second messengers and intermediates in lipid metabolism and have various roles in pathways of pain induction and maintenance [16,35,36]. LPC is a subgroup of the lysophospholipid family and mainly derived from PC by phospholipase A_2_ (PLA_2_) [37]. LPC, as a proalgesic mediator, induces pain by increasing nitric oxide synthase in dorsal root ganglion (DRG) or activating the acid-sensing ion channel 3 [33,38]. LPC can also be hydrolyzed by autotaxin (ATX), a secreted exoenzyme with lysophospholipase D activity, to generate LPA, a compound that has been reported to be profoundly involved in the initiation and maintenance of chronic pain [17,39]. LPA mediates the activation of ion channels such as TRPV1 in DRG neurons to induce central sensitization [40]. Through the activation of G protein-coupled receptors, LPA also mediates the demyelination of central neurons, which is associated with the development of chronic pain [41,42]. The activation of ATX-LPA axis signaling pathway is likely involved in the maintenance of pain, which could have led to the decreased level of LPC class in persistent MSMP patients observed in our study. Future studies on serum LPA level and ATX activity in persistent MSMP are needed to provide more information.

The ratio of LPC to PC has been reported to be associated with inflammation in severe OA and RA [43,44]. Consistent with one previous cross-sectional study [22], no association between the ratio of LPC to PC and MSMP was observed in the current study. Furthermore, our longitudinal analysis found no associations between ratio of LPC to PC and persistent MSMP. On the contrary, Zhang et al. [45] reported the increased ratio of LPC to PC, driven by significantly higher LPC and lower PC levels in knee OA patients than in healthy controls, as an important marker for predicting advanced knee OA. This may suggest that persistent MSMP may be largely driven by central sensitization other than inflammation. 

There are several limitations in this study. First, the majority of lipid species were intermediate metabolites. The key downstream products, such as LPA, were not quantified in this study. However, this study is the first to explore lipidomic profiles in persistent MSMP. The identified lipid signatures of persistent MSMP shed light on underlying pathogenesis of persistent MSMP and provide robust data for future studies on its biological pathways. Second, lipidomics was only performed on serum samples at a 2.6-year follow-up, so we are unable to examine whether variations of lipid species from 2.6 to 10.7 years are associated with persistent MSMP. Third, this study included 536 subsamples with MSMP data at each follow-up time-point and blood samples available from the Tasmanian Older Adult Cohort study (TASOAC) cohort. However, there were no differences in the characteristics between those included in this study and the whole cohort, suggesting minimization of the bias. Fourth, pain was assessed via a simple questionnaire (yes/no); therefore, we are unable to investigate whether lipids are associated with other pain features such as pain duration and frequency in this study. Fifth, given the nature of observational study, we are unable to determine whether the way to modify lipids level by diet, hormonal regulation and exercise management can affect MSMP; thus, future trials are needed to examine whether interventions to modify lipid levels can affect MSMP.

## 4. Materials and Methods

### 4.1. Study Participants

The study participants were derived from the TASOAC, which is a prospective, population-based cohort study. The TASOAC study recruited 1100 participants aged 50–80 years who were randomly selected from the electoral roll in southern Tasmania (population *n* = 229,000), Australia, between 2002–2004. A total of 1099 participants had their baseline examinations including questionnaire, general interview, clinical assessment and blood tests. Participants were subsequently followed up at 2.6, 5.1 and 10.7 years. Fasting blood samples collected at the 2.6-year follow-up were used for the lipidomics assay due to depletion of baseline blood samples. A total of 536 participants with MSMP data at each follow-up time point and blood samples available were included in the study. The TASOAC study was approved by the Southern Tasmanian Health and Medical Human Research Ethics Committee (Ref. no: H0006488) and written informed consent was obtained from all participants. 

### 4.2. Demographic and Medical Information Collection

Date of birth and sex were self-reported and age at the recruitment was calculated. Height and weight were measured at 2.6-year follow-up and BMI (kg/m^2^) was calculated. Physical activity at 2.6-year follow-up was measured by steps per day for 7 consecutive days using a pedometer (Omron HJ-003 and HJ-102; Omron Healthcare, Kyoto, Japan). Comorbidities including diabetes, heart attack, hypertension, thrombosis, asthma, bronchitis/emphysema, hyperthyroidism, hypothyroidism and RA at 2.6-year follow-up were self-reported. OA in the neck, back, hands, shoulders, hips, knees and feet at 2.6-year follow-up was diagnosed by physicians.

### 4.3. MSMP Assessment 

Pain at any of the seven anatomical sites including neck, shoulder, hand, back, hip, knee and foot was self-reported using a pain questionnaire, and total number of painful sites was calculated. In accordance with the 2016 widespread pain definition (WP2016) [46], MSMP was defined as having ≥4 painful sites. Persistent MSMP was then defined as having MSMP at 2.6-, 5.1- and 10.7-year follow-up, and non-persistent MSMP was defined as not having MSMP during at least one of the follow-ups [47].

### 4.4. Lipidomic Profiling

Blood samples were collected after at least 8 h fasting using the white top Greiner tubes with gel and left at room temperature for 10 min. The tubes were inverted a few times by hand to help activate the clotting process. Then, blood samples were centrifuged at 2500 rpm for 5 min, and the serum was then transferred into microcentrifuge tubes and stored at −80 °C until analysis. Serum samples were randomized, and lipids were extracted using the butanol/methanol method as described previously [48]. Targeted lipidomic analysis was performed on an Agilent 6495C QQQ mass spectrometer coupled with an Agilent 1290 series HPLC system and a ZORBAX eclipse plus C18 column (Agilent, Santa Clara, CA, USA) in positive and negative ion mode as described previously [48]. Technical quality controls (TQCs) and pooled serum QCs (PQCs) were used to monitor the overall performance of the liquid chromatography-tandem mass spectrometry (LC-MS/MS) analysis and experimental issues during lipid extraction. NIST1950 samples representing the “population” lipidomic profile were used to facilitate future alignment with other studies. Chromatographic peaks for each lipid were integrated based on dynamic multiple reaction monitoring (dMRM) ion pairs and retention time using the MassHunter software (Agilent). Lipid concentrations were calculated by relating the area under the chromatogram for each lipid species to the corresponding internal standard. Correction factors were applied to adjust for different response factors where these were known. Mean and median coefficients of variation (CVs) of all QCs were ≤11.7%, and those of PQCs were 9.4% and 7.6%, respectively.

### 4.5. Statistical Analysis

Normality of distribution was tested with the Shapiro–Wilk test. Age, BMI, steps per day were compared with the Mann–Whitney U test. All lipid and lipid class concentrations were natural log transformed for normalization and compared with independent sample Student’s *t*-test. The ratio of LPC to PC was calculated using original concentrations of LPC and PC classes and then natural log transformed. Sex distribution and prevalence of comorbidities were compared with Chi-squared test or Fisher’s exact test wherever appropriate. The covariates included in multivariable analyses were age, sex, BMI, physical activity and presence of any comorbidities (OA, RA, emphysema, and diabetes). Age, sex, BMI and physical activity were correlated with MSMP or lipidomic metabolites, or both, based on pervious literature [48,49,50]. OA is the most common cause of musculoskeletal pain [51]. We also compared the difference in all 10 comorbidities between the MSMP and non-MSMP group, and found the prevalence of OA, RA, emphysema and diabetes was higher in the MSMP group. These four comorbidities have also been reported to be associated with lipids in the existing literature [52,53,54,55]. Therefore, the presence of any of these four comorbidities was included as a covariate in the multivariable analyses. Logistic regression was utilized to identify lipid species and classes associated with MSMP at 2.6-year follow-up and those associated with persistent MSMP, with adjustment for potential confounders including age, sex, BMI, physical activity and presence of any comorbidities. The Benjamini–Hochberg method [56] was used to control multiple testing for lipid and lipid class concentrations. For all other variables, the significance level was defined at α ≤ 0.05. All analyses were performed in R Studio with R version 3.6.3. Visualizations of the results were done with ggplot2 R package.

## 5. Conclusions

This study identified three novel lipid signatures of persistent MSMP, suggesting that lipid metabolism is involved in the pathogenesis of persistent pain. 

## Figures and Tables

**Table 1:** Participants’ characteristics *.

	Total (*n* = 530)	Persistent MSMP (*n* = 112)	Non-Persistent MSMP (*n* = 418)	*p* Value
Age (years)	61.54 ± 6.57	61.71 ± 6.65	61.49 ± 6.56	0.80
BMI (kg/m^2^)	27.73 ± 4.54	28.13 ± 4.73	27.62 ± 4.48	0.31
Females (%)	50	67	46	<0.001
Physical activity (steps per day)	7865.21 ± 3253.78	7234.72 ± 3009.63	8034.60 ± 3299.35	0.04
Comorbidities (%)	40	70	31	<0.001
OA (%)	37	65	30	<0.001
RA (%)	3	5	3	0.23
Emphysema (%)	3	7	2	0.01
Diabetes (%)	2	4	1	0.23

MSMP: multisite musculoskeletal pain; BMI: body mass index; OA: osteoarthritis; RA: rheumatoid arthritis. * Values are the mean ± SD unless indicated otherwise. *p* values were obtained by Mann–Whitney U test, Chi-squared test or Fisher’s exact test wherever appropriate.

**Table 2:** Lipid species and class associated with MSMP at 2.6-year follow-up.

	Univariable				Multivariable *			
	*p* Value	OR	2.5% CI	97.5% CI	*p* Value	OR	2.5% CI	97.5% CI
**Lipid species**								
SM(38:3) (a)	3.30 × 10^−4^	4.45	1.99	10.19	6.12 × 10^−2^	2.61	0.96	7.23
HexCer(d18:1/24:0)	3.60 × 10^−3^	0.38	0.20	0.73	7.50 × 10^−2^	0.51	0.25	1.07
SM(40:4)	3.91 × 10^−3^	4.79	1.67	14.04	1.59 × 10^−1^	2.55	0.70	9.44
AC(26:0)	5.27 × 10^−3^	0.42	0.23	0.77	6.84 × 10^−1^	0.86	0.41	1.80
**Lipid class**								
HexCer	2.21 × 10^−2^	0.44	0.21	0.88	1.21 × 10^−1^	0.53	0.24	1.18

MSMP: multisite musculoskeletal pain; OR: odds ratio; CI: confidence interval; HexCer: monohexosylceramide; SM: sphingomyelin; AC: acylcarnitine. *p* values were obtained by univariable or multivariable logistic regression. * Adjusted for age, sex, body mass index, physical activity and presence of at least one of the four comorbidities.

**Table 3:** Lipid species associated with persistent MSMP.

	Univariable				Multivariable *			
	*p* Value	OR	2.5% CI	97.5% CI	*p* Value	OR	2.5% CI	97.5% CI
HexCer(d18:1/22:0)	3.46 × 10^−4^	0.26	0.12	0.54	7.71 × 10^−3^	0.33	0.14	0.74
HexCer(d18:1/24:0)	4.55 × 10^−5^	0.20	0.09	0.43	2.15 × 10^−3^	0.25	0.10	0.60
LPC(18:1) [sn1]	6.60 × 10^−4^	0.23	0.09	0.53	1.46 × 10^−2^	0.31	0.12	0.78
LPC(15-MHDA) [sn1] [104_sn1]	6.48 × 10^−4^	0.32	0.16	0.61	7.95 × 10^−3^	0.36	0.17	0.76
LPC(18:2) [sn1]	6.53 × 10^−4^	0.28	0.13	0.58	4.70 × 10^−2^	0.41	0.17	0.98
LPC(18:2) [sn2]	4.06 × 10^−4^	0.21	0.09	0.50	1.91 × 10^−2^	0.30	0.11	0.82
AC(26:0)	2.19 × 10^−4^	0.25	0.12	0.52	5.01 × 10^−2^	0.41	0.17	0.99

MSMP: multisite musculoskeletal pain; OR: odds ratio; CI: confidence interval; HexCer: monohexosylceramide; LPC: lysophosphatidylcholine; MHDA: methylhexadecanoic acid; AC: acylcarnitine. *p* values were obtained by univariable or multivariable logistic regression. * Adjusted for age, sex, body mass index, physical activity and presence of at least one of the four comorbidities.

**Table 4:** Lipid classes associated with persistent MSMP.

	Univariable				Multivariable *			
	*p* Value	OR	2.5% CI	97.5% CI	*p* Value	OR	2.5% CI	97.5% CI
HexCer	2.23 × 10^−4^	0.20	0.08	0.46	2.02 × 10^−3^	0.22	0.08	0.57
LPC	1.02 × 10^−3^	0.18	0.06	0.49	2.50 × 10^−2^	0.27	0.08	0.84
LPC(O)	2.35 × 10^−3^	0.22	0.08	0.58	6.88 × 10^−2^	0.36	0.12	1.07

MSMP: multisite musculoskeletal pain; OR: odds ratio; CI: confidence interval; HexCer: monohexosylceramide; LPC: lysophosphatidylcholine; LPC(O): lysoalkylphosphatidylcholine. *p* values were obtained by univariable or multivariable logistic regression. * Adjusted for age, sex, body mass index, physical activity and presence of at least one of the four comorbidities.

## Data Availability

The data presented in this study are available on request from the corresponding author. The data are not publicly available due to privacy restrictions.

## Supplementary Materials

The following supporting information can be downloaded at: https://www.mdpi.com/article/10.3390/metabo12030206/s1, Table S1: Serum lipid species associated with MSMP at 2.6-year follow-up, Table S2: Serum lipid classes associated with MSMP at 2.6-year follow-up, Table S3: Serum lipid species associated with persistent MSMP, Table S4: Serum lipid classes associated with persistent MSMP.
